# The nasal cavity microbiota of healthy adults

**DOI:** 10.1186/2049-2618-2-27

**Published:** 2014-08-11

**Authors:** Christine M Bassis, Alice L Tang, Vincent B Young, Melissa A Pynnonen

**Affiliations:** 1 Department of Internal Medicine, Division of Infectious Diseases, University of Michigan, 1510 MSRB I SPC 5666, 1150 W. Medical Center Dr, Ann Arbor, MI 48109-5666, USA; 2 Department of Otolaryngology, University of Cincinnati, Medical Sciences Building, 231 Albert Sabin Way, Cincinnati, OH 45267-0528, USA; 3 Department of Otolaryngology, University of Michigan, 1500 E. Medical Center Drive, Taubman Center 1904, Ann Arbor, MI 48109-0312, USA

**Keywords:** Nasal cavity, Oral cavity, Tongue, Buccal mucosa, Microbiota, Culture-independent

## Abstract

**Background:**

The microbiota of the nares has been widely studied. However, relatively few studies have investigated the microbiota of the nasal cavity posterior to the nares. This distinct environment has the potential to contain a distinct microbiota and play an important role in health.

**Results:**

We obtained 35,142 high-quality bacterial 16S rRNA-encoding gene sequence reads from the nasal cavity and oral cavity (the dorsum of the tongue and the buccal mucosa) of 12 healthy adult humans and deposited these data in the Sequence Read Archive (SRA) of the National Center for Biotechnology Information (NCBI) (Bioproject: PRJNA248297). In our initial analysis, we compared the bacterial communities of the nasal cavity and the oral cavity from ten of these subjects. The nasal cavity bacterial communities were dominated by Actinobacteria, Firmicutes, and Proteobacteria and were statistically distinct from those on the tongue and buccal mucosa. For example, the same Staphylococcaceae operational taxonomic unit (OTU) was present in all of the nasal cavity samples, comprising up to 55% of the community, but Staphylococcaceae was comparatively uncommon in the oral cavity.

**Conclusions:**

There are clear differences between nasal cavity microbiota and oral cavity microbiota in healthy adults. This study expands our knowledge of the nasal cavity microbiota and the relationship between the microbiota of the nasal and oral cavities.

## Background

Bacterial communities play important roles in the health of their hosts, including roles in immune system development [1], nutrition [2], and resistance to infection [3]. In this study, we compared the nasal cavity microbiota and the oral cavity microbiota from healthy adult humans. Until recently, the bacterial community of the healthy human nasal cavity had not been characterized by culture-independent methods [4,5]. However, many studies have characterized the microbiota of the healthy human nares [6-11], which are adjacent and anterior to the nasal cavity. *Staphylococcus aureus* carriage in the nares is linked to increased risk of *S. aureus* infection in other body sites [12,13]. Further, antagonism by and competition with other members of the nares microbiota seem to influence *S. aureus* nares carriage [12]. Although adjacent to the nares, the nasal cavity is distinct from the nares with a different type of epithelium, a non-keratinized stratified squamous epithelium that transitions to a typical respiratory epithelium—ciliated pseudostratified columnar epithelial cells and mucus-producing goblet cells [14]. In contrast, the nares have a keratinized, stratified squamous epithelium with hairs and sebaceous glands. Relatively few studies have investigated the bacterial community composition of the nasal cavity in healthy humans. In this study, we sought to expand our knowledge of the healthy human nasal cavity microbiota and compare the nasal cavity microbiota to the oral cavity microbiota in the same subjects.

## Methods

### Subject recruitment and characteristics

This study was approved by the University of Michigan Institutional Review Board. All subjects provided written informed consent. Twelve adults patients were recruited from a tertiary care otolaryngology clinic (Additional file 1: Table S1). Exclusion criteria were patients who had acute or chronic sinusitis and patients who were taking antibiotics or oral steroids for any reason.

### Sampling

4N6 DNA flocked swabs (Cat. No. 3520CA, Copan Diagnostics Inc., Murrieta, CA, USA) were used to sample all sites. The nasal cavity was sampled by inserting the swab into the nasal passage between the septum and middle turbinate, taking care to avoid contact to the nares. The dorsum of the tongue and buccal mucosa were sampled with separate swabs. The samples were transferred directly into the Eppendorf tubes provided with the swab and stored on ice and then at −20°C until DNA isolation.

### DNA isolation

DNA was isolated from the swabs with a PowerSoil DNA isolation kit (Mo Bio Laboratories, Inc., Carlsbad, CA, USA) according to the manufacturer's instructions except that 2 min of bead beating using the ‘Homogenize’ setting of a Mini-BeadBeater-8 (Biospec Products, Bartlesville, OK, USA) was done in place of 10 min of vortexing.

### Primary PCR amplification, pooling, and sequencing

We based our protocol for amplifying and preparing libraries of the V5V3 region of the 16S rRNA-encoding gene on HMP 16S Protocol Version 4.2 (http://www.hmpdacc.org/doc/16S_Sequencing_SOP_4.2.2.pdf). Each 20 μl polymerase chain reaction (PCR) reaction contained 2 μl AccuPrime PCR Buffer II (Invitrogen, Carlsbad, CA, USA), 0.15 μl AccuPrime Taq DNA Polymerase High Fidelity (Invitrogen), 0.2 μM primer A (CCATCTCATCCCTGCGTGTCTCCGACTCAGXXXXX**CCGTCAATTCMTTTRAGT**), 0.2 μM primer B (CCTATCCCCTGTGTGCCTTGGCAGTCTCAG**CCTACGGGAGGCAGCAG**), and 1 μl DNA for the oral cavity samples or 15.45 μl DNA for the nasal samples. The bold portions of primer A and primer B are 926R and 357 F, respectively. The region of primer A represented by XXXXX is the 5–10 nucleotide barcode sequence. The remainder of primer A and primer B are the A adapter sequence and the B adapter sequence, respectively, required for emPCR and 454 sequencing. The PCR was run for 2 min at 95°C followed by 30 cycles of 95°C for 20 s, 50°C for 30 s, and 72°C for 5 min. The PCR products were purified with AMPure XP (Agencourt Bioscience Corporation, Beckman Coulter, Inc., Beverly, MA, USA) according to the manufacturer's instructions except 0.6× the amplicon volume (10.8 μl) of beads was used rather than 1.2× in order to remove more of the small products. The purified PCR products were quantified with a Quant-iT PicoGreen dsDNA kit (Invitrogen) according to the manufacturer's instructions and combined into a pool with equal amounts of each amplicon. The pool was then purified with AMPure XP (Agencourt Bioscience Corporation) according to the manufacturer's instructions except the volume of beads was 0.6× the pool volume. The pool was quantified with a Library Quantification Kit for Roche 454 GS Titanium (Kapa Biosystems, Inc., Wilmington, MA, USA). A Junior emPCR (454 Life Sciences, Roche, Branford, CT, USA) was performed, and 454 sequencing was done on a GS Junior (454 Life Sciences) according to manufacturer's instructions.

### Sequence processing

Sequences were processed with mothur v.1.28.0 according to the Schloss SOP of November 27, 2012 [15,16]. In summary, the sff file was input to sffinfo, trim.flows was run allowing 1 mismatch in the barcode and 2 mismatches in the 926R region of the primer, and sequencing error was reduced with shhh.flows. With trim.seqs, barcode and primer sequences were removed and all sequences less than 200 bases or with homopolymers longer than eight nucleotides were discarded. The sequences were aligned to the Silva reference alignment [17,18]. In order to compare sequences over the same region of the alignment, we set the end position at 27659 and chose a start position that was met by 95% of the sequences. With pre.cluster, sequences within two base pairs were merged. Chimeras were identified with chimera.uchime [19] and removed. The sequences were classified using a modified form of RDP training set version 9 (trainset9_032012.pds.tax and trainset9_032012.pds.fasta) [20]. Sequences classified as Chloroplast, Mitochondria, Archaea, Eukaryota, or unknown kingdom were removed.

### Sequence analysis

For our initial analysis, we included subjects only if sequences were obtained for a complete set of samples (buccal mucosa, tongue, and nasal cavity). Therefore, in order to maximize the number of subjects included in the study, we decided to subsample 269 sequences from each sample. So, subjects were included in our initial analysis only if at least 269 sequences were obtained from all three sites (nasal cavity, buccal mucosa, and dorsal side of tongue). A distance matrix made with dist.seqs was used with the average neighbor algorithm to group sequences into operational taxonomic units (OTUs) with the cluster command. OTUs defined as 3% different were used for further analysis. The make.shared command was used to produce a table (shared file) of the number of sequence reads assigned to each OTU in each sample. The shared file was used to calculate *θ* (1 − *θ* similarity index), a metric of community dissimilarity that takes the relative abundances of both shared and non-shared OTUs into account [21]. Principle coordinates analysis (PCoA) was used to visualize the *θ* distance matrix, and analysis of molecular variance (AMOVA) [22] was used to test the statistical significance of the differences between bacterial communities of different groups (i.e., nasal cavity communities versus buccal mucosa communities).

## Quality assurance

To ensure that the source of bacterial sequences was not the swab itself or the DNA isolation reagents, PCR was performed on DNA isolated from an unused swab. To confirm that the PCR reagents were not the source of bacterial sequences, PCR of the no-template control was performed. Neither of these control PCRs yielded products visible on a gel, indicating that there was minimal contamination from the swab or reagents.

## Initial findings

### Sequences obtained

After sequence processing, we obtained a total of 35,142 high-quality bacterial 16S rRNA-encoding gene sequence reads from 35 buccal mucosa, tongue (dorsal side), and nasal cavity samples from 12 subjects with a mean of 1,115 sequences per sample (Additional file 1: Table S1). The mean sequence length after sequence processing was 268 bases. The number of sequences obtained per sample from the buccal mucosa microbiota ranged from 715–1,684. The number of sequences obtained per sample from the tongue microbiota ranged from 519–1,597. The number of sequences obtained per sample from the nasal cavity microbiota ranged from 1–1,595. The sff files from which the processed sequences were generated were submitted to the SRA (Bioproject:PRJNA248297) except for the sff that yielded only one sequence (nasal cavity L) (Additional file 1: Table S1).

### Nasal cavity bacterial communities were distinct from the bacterial communities on the tongue and buccal mucosa

**Figure 1:**
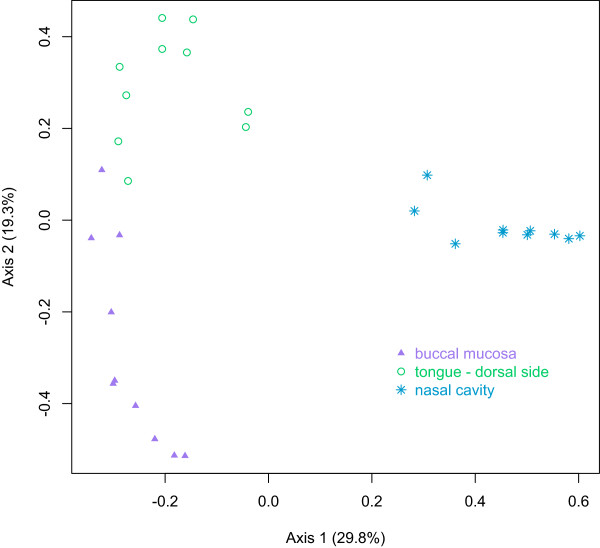
**Principal coordinates analysis of nasal cavity, buccal mucosa and tongue microbiota.** Principal coordinates analysis was performed on a matrix of *θ* distances between all samples. OTUs were defined as 3% different. Axis 1, representing 29.8% of the variance, and axis 2, representing 19.3% of the variance, are displayed.

For our initial analysis, we subsampled 269 sequences from each sample following sequence processing. Ten healthy adult subjects had at least 269 sequences from each sampling site following sequence processing (nasal cavity, dorsum of the tongue, and buccal mucosa) and were included in the analysis (Additional file 1: Table S1). As illustrated by PCoA of *θ* dissimilarities, the bacterial communities clustered based on body site, with the nasal cavity bacterial communities more similar to each other than to the oral cavity bacterial communities (Figure 1). Based on *θ* dissimilarities, the differences between the bacterial communities of the nasal cavity and each oral cavity subsite were statistically significant (AMOVA *p* value <0.001 for each comparison). Additionally, although the bacterial communities of the two sites in the oral cavity were more similar to each other than to the nasal cavity communities, based on *θ* dissimilarities, the bacterial communities from the tongue and the buccal mucosa were distinct (AMOVA *p* value <0.001).

### Composition of the nasal cavity microbiota

**Figure 2:**
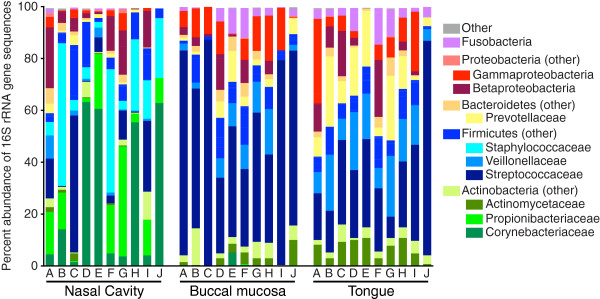
**Bacterial community composition of the nasal cavity, buccal mucosa, and tongue.** Two hundred sixty-nine sequences were subsampled from each sample. The sequences were then classified as described in the materials and methods, and the relative abundances of sequences classified to major taxonomic groups are displayed.

In our study and other recent studies [4,5], the bacterial communities of the nasal cavities were dominated by Actinobacteria, Firmicutes, and in some cases, Proteobacteria (Figure 2). Corynebacteriaceae and Propionibacteriaceae were the most prevalent families of Actinobacteria in the nasal cavity (Figure 2). Between subjects, the levels of Corynebacteriaceae varied from 1.5% to 62.8%, and the levels of Propionibacteriaceae varied from 0.4% to 42.4% (Figure 2, Additional file 2: Table S2). Actinobacteria were present at lower levels in the communities at both oral cavity sites, but they were from the families Micrococcaceae and Actinomycetaceae. Corynebacteriaceae composed over 1% of the community in only one oral cavity sample. Propionibacteriaceae was undetectable in most oral cavity samples, and when it was detected, it never exceeded 1% of the community.

Another striking difference between the nasal cavity and oral cavity communities was the composition of the Firmicutes. The same Staphylococcaceae OTU was present in the nasal cavity samples of all subjects, ranging from 2.2%–55.0% of the community, while Staphylococcaceae were not detected in the oral cavity communities (Additional file 2: Table S2). It was not possible to distinguish *Staphylococcus aureus* and *Staphylococcus epidermidis* in the region of the 16S rRNA gene covered by our sequences. Generally, in the oral cavity, Streptococcaceae was the most abundant Firmicutes family, and in several subjects, the most abundant family overall. Veillonellaceae was also more abundant in the oral cavity.

The overall levels of Betaproteobacteria were not significantly different between nasal cavity communities and oral cavity communities (Kruskal-Wallis test, *p* value = 0.4557). However, the composition of the Betaproteobacteria differed between the nasal cavity and the oral cavity: all three sites included Neisseriaceae at levels that were not statistically different (Kruskal-Wallis test, *p* value = 0.4543), but the nasal cavity contained higher levels of Comamonadaceae (Kruskal-Wallis test, *p* value = 0.0002) and *Burkholderiales incertae sedis* (Kruskal-Wallis test, *p* value = 0.0004).

## Future directions

With its proximity to the sinuses—the maxillary sinuses communicate with the nasal cavity through 1 to 2-mm ostia—the nasal cavity microbiota might be a useful proxy for the less accessible sinus microbiota. Signatures of sinusitis in the sinus microbiota have recently been identified [23]. To determine if specific changes in the nasal cavity microbiota also accompany sinusitis and could be used as an indicator of sinus infections, it will be necessary to sample both the nasal cavity and the sinus in the same subjects with and without sinusitis.

## Availability of supporting data

The sff files have been deposited in the SRA (Bioproject: PRJNA248297).

## Abbreviations

OTU: Operational taxonomic unit; PCoA: Principal coordinates analysis; PCR: Polymerase chain reaction.

## Competing interests

The authors declare that they have no competing interests.

## Authors’ contributions

The study was designed by ALT, VBY, and MAP. ALT and MAP did the sample collection. DNA isolation was done by ALT. Sequencing library preparation and sequence analysis were performed by CMB. CMB and MAP wrote the manuscript. CMB, ALT, VBY, and MAP edited the manuscript. All authors read and approved the final manuscript.

## Supplementary Material

Additional file 1: Table S1 Number of sequences obtained from each sample and subject characteristics. Click here for file

Additional file 2: Table S2 Relative abundances of bacterial families in each sample based on classification of 16S rRNA-encoding gene sequence reads. Click here for file
