# Supplementary material for: The nasal cavity microbiota of healthy adults
Source: Microbiome. 2014 Aug 11;2:27. doi: 10.1186/2049-2618-2-27 (PMC4138944; doi:10.1186/2049-2618-2-27)
Supplement: Additional file 2: Table S2 — Relative abundances of bacterial families in each sample based on classification of 16S rRNA-encoding gene sequence reads. [file 2049-2618-2-27-S2.pdf]

Supplemental Table 2

	Nasal Cavity									
	A	B	C	D	E	F	G	H	I	J
**Actinobacteria**										
Corynebacteriaceae	4.5%	14.1%	1.5%	63.2%	60.6%	4.8%	3.7%	55.4%	4.1%	62.8%
Propionibacteriaceae	16.4%	14.1%	0.4%	1.5%	21.2%	18.6%	42.4%	3.3%	13.8%	9.7%
Actinomycetaceae	1.9%	1.1%	3.0%	0.4%	0.4%	0.7%	0.4%	0.0%	0.0%	0.0%
Micrococcaceae	0.4%	0.0%	0.0%	0.7%	0.0%	0.0%	0.4%	0.4%	0.0%	0.0%
Other	3.0%	1.1%	0.4%	5.2%	0.4%	0.0%	1.9%	0.0%	10.8%	0.0%
**Bacteroidetes**										
Prevotellaceae	3.7%	0.7%	3.7%	0.7%	1.5%	1.5%	0.7%	1.1%	0.0%	0.0%
Flavobacteriaceae	4.8%	3.0%	0.0%	0.7%	0.0%	0.7%	2.2%	0.0%	0.4%	0.0%
Porphyromonadaceae	1.1%	0.0%	0.4%	0.0%	0.0%	0.4%	0.0%	0.0%	0.0%	0.0%
Other	0.0%	0.0%	1.1%	0.0%	0.0%	0.0%	0.7%	0.0%	0.0%	0.0%
**Firmicutes**										
Streptococcaceae	16.0%	1.1%	53.2%	1.1%	6.3%	3.7%	11.9%	1.1%	27.9%	0.7%
Veillonellaceae	8.9%	0.0%	1.9%	0.7%	3.7%	1.1%	0.4%	0.4%	0.7%	0.0%
Staphylococcaceae	5.2%	55.0%	4.1%	4.5%	2.2%	47.6%	3.3%	27.5%	14.9%	23.0%
Bacillales Family XI. Incertae Sedis	0.0%	0.0%	0.0%	0.0%	0.0%	1.5%	0.0%	0.0%	0.0%	0.0%
Unclassified Lactobacillales	0.0%	0.0%	0.0%	0.0%	0.0%	0.0%	1.1%	0.0%	0.4%	0.0%
Lachnospiraceae	1.9%	0.7%	1.9%	0.0%	1.1%	0.0%	1.1%	0.0%	0.7%	0.0%
Clostridiales Family XI. Incertae Sedis	0.4%	3.0%	0.0%	1.1%	0.0%	3.0%	3.3%	10.4%	4.8%	0.0%
Peptostreptococcaceae	0.0%	0.0%	17.5%	0.0%	0.0%	0.0%	0.0%	0.0%	4.1%	0.0%
Carnobacteriaceae	0.0%	0.4%	0.0%	16.4%	0.0%	0.0%	0.4%	0.0%	0.0%	3.7%
Other	1.1%	0.0%	1.9%	0.0%	0.4%	0.0%	0.4%	0.0%	2.2%	0.0%
**Fusobacteria**										
Fusobacteriaceae	1.1%	0.0%	0.0%	0.0%	0.0%	0.4%	0.0%	0.0%	0.0%	0.0%
Leptotrichiaceae	0.4%	0.0%	0.7%	0.0%	0.7%	0.0%	0.4%	0.0%	0.0%	0.0%
**Proteobacteria**										
**Betaproteobacteria**										
Neisseriaceae	2.2%	0.7%	0.7%	0.0%	0.0%	11.5%	0.0%	0.4%	13.4%	0.0%
Comamonadaceae	13.0%	2.2%	1.5%	1.1%	0.4%	1.5%	10.8%	0.0%	0.0%	0.0%
Burkholderiales_incertae_sedis	8.2%	1.1%	3.0%	0.4%	0.4%	0.4%	5.9%	0.0%	0.7%	0.0%
Other	0.0%	0.4%	0.0%	0.4%	0.0%	0.0%	0.4%	0.0%	0.0%	0.0%
**Gammaproteobacteria**										
Pasteurellaceae	0.0%	0.0%	0.4%	0.0%	0.0%	1.9%	1.9%	0.0%	0.7%	0.0%
Moraxellaceae	1.5%	0.0%	1.1%	1.1%	0.4%	0.0%	4.5%	0.0%	0.4%	0.0%
Other	2.6%	0.4%	1.9%	0.0%	0.0%	0.4%	0.4%	0.0%	0.0%	0.0%
**Other Proteobacteria**	1.5%	0.0%	0.0%	0.4%	0.4%	0.4%	1.5%	0.0%	0.0%	0.0%
**Other Phyla**	0.4%	0.7%	0.0%	0.4%	0.0%	0.0%	0.0%	0.0%	0.0%	0.0%

	Buccal Mucosa									
	A	B	C	D	E	F	G	H	I	J
**Actinobacteria**										
Corynebacteriaceae	0.0%	0.0%	0.0%	0.0%	5.2%	0.4%	0.0%	0.0%	0.0%	0.0%
Propionibacteriaceae	0.0%	0.0%	0.0%	0.0%	0.0%	0.4%	0.0%	0.0%	0.0%	0.4%
Actinomycetaceae	1.5%	0.4%	0.0%	3.0%	3.3%	4.5%	3.0%	3.0%	0.0%	9.7%
Micrococcaceae	2.2%	14.1%	0.0%	1.9%	0.0%	1.9%	5.6%	5.6%	0.4%	5.6%
Other	0.4%	0.0%	0.0%	0.0%	2.6%	0.4%	0.7%	0.4%	0.0%	0.0%
**Bacteroidetes**										
Prevotellaceae	1.1%	1.1%	0.0%	6.3%	11.9%	3.7%	2.2%	3.0%	0.0%	5.9%
Flavobacteriaceae	0.0%	0.4%	0.0%	2.2%	4.8%	3.0%	0.4%	0.4%	0.0%	0.0%
Porphyromonadaceae	1.1%	1.5%	0.0%	0.7%	0.7%	1.9%	2.6%	1.5%	0.0%	0.4%
Other	0.0%	0.4%	0.0%	1.5%	0.0%	0.4%	0.4%	0.0%	0.0%	0.0%
**Firmicutes**										
Streptococcaceae	79.2%	54.3%	87.4%	29.7%	43.1%	30.5%	50.2%	34.6%	79.2%	67.7%
Veillonellaceae	2.6%	2.2%	0.0%	7.4%	8.9%	9.3%	11.9%	17.5%	0.0%	2.2%
Staphylococcaceae	0.0%	0.0%	0.0%	0.0%	0.0%	0.0%	0.0%	0.0%	0.0%	0.0%
Bacillales Family XI. Incertae Sedis	0.0%	7.8%	1.1%	4.1%	0.0%	6.7%	3.0%	1.5%	2.2%	1.1%
Unclassified Lactobacillales	0.7%	1.5%	0.0%	1.9%	1.1%	4.8%	3.0%	4.1%	1.1%	0.0%
Lachnospiraceae	0.0%	0.7%	0.0%	1.1%	1.5%	3.0%	0.4%	2.6%	0.0%	0.7%
Clostridiales Family XI. Incertae Sedis	0.0%	0.0%	0.0%	0.4%	3.0%	0.0%	0.0%	0.0%	0.4%	0.4%
Peptostreptococcaceae	0.0%	0.4%	0.0%	0.7%	0.4%	0.0%	0.0%	0.0%	0.0%	0.7%
Carnobacteriaceae	0.0%	0.0%	0.0%	0.4%	0.0%	0.0%	0.0%	0.0%	0.0%	0.0%
Other	0.4%	0.4%	0.0%	7.1%	2.2%	0.4%	0.0%	0.4%	0.0%	1.1%
**Fusobacteria**										
Fusobacteriaceae	1.5%	0.4%	0.0%	4.8%	9.7%	9.3%	2.6%	0.7%	0.4%	2.6%
Leptotrichiaceae	0.0%	0.0%	0.0%	0.4%	0.0%	2.2%	0.4%	2.2%	0.0%	0.0%
**Proteobacteria**										
**Betaproteobacteria**										
Neisseriaceae	2.2%	3.3%	1.1%	12.3%	0.7%	8.9%	0.0%	3.7%	0.0%	0.0%
Comamonadaceae	0.0%	0.0%	0.0%	0.0%	0.0%	0.0%	0.0%	0.0%	0.0%	0.0%
Burkholderiales_incertae_sedis	0.4%	0.0%	0.0%	0.0%	0.0%	0.0%	0.0%	0.0%	0.0%	0.0%
Other	0.4%	0.0%	0.0%	1.5%	0.0%	0.0%	0.0%	1.5%	0.0%	0.0%
**Gammaproteobacteria**										
Pasteurellaceae	6.3%	11.2%	10.4%	11.5%	0.4%	8.2%	13.4%	17.5%	16.4%	0.4%
Moraxellaceae	0.0%	0.0%	0.0%	1.1%	0.0%	0.0%	0.0%	0.0%	0.0%	0.0%
Other	0.0%	0.0%	0.0%	0.0%	0.0%	0.0%	0.0%	0.0%	0.0%	0.0%
**Other Proteobacteria**	0.0%	0.0%	0.0%	0.0%	0.0%	0.4%	0.0%	0.0%	0.0%	0.4%
**Other Phyla**	0.0%	0.0%	0.0%	0.0%	0.4%	0.0%	0.4%	0.0%	0.0%	0.7%

	Tongue - dorsal side									
	A	B	C	D	E	F	G	H	I	J
**Actinobacteria**										
Corynebacteriaceae	0.0%	0.0%	0.0%	0.0%	0.0%	0.0%	0.0%	0.0%	0.0%	0.0%
Propionibacteriaceae	0.4%	0.0%	0.0%	0.0%	0.0%	0.0%	0.4%	0.0%	0.0%	0.0%
Actinomycetaceae	7.8%	3.0%	9.3%	10.0%	10.8%	3.0%	7.4%	10.8%	4.8%	0.7%
Micrococcaceae	1.9%	0.0%	6.7%	0.7%	1.5%	2.6%	1.5%	3.7%	4.5%	3.3%
Other	0.0%	2.2%	0.0%	0.0%	2.6%	0.0%	1.5%	0.4%	0.4%	0.0%
**Bacteroidetes**										
Prevotellaceae	7.4%	27.1%	8.6%	18.6%	21.6%	3.3%	24.9%	12.6%	1.5%	3.3%
Flavobacteriaceae	0.0%	0.4%	0.4%	0.4%	0.0%	0.4%	1.1%	0.0%	0.0%	0.0%
Porphyromonadaceae	2.2%	1.9%	0.7%	0.4%	0.0%	1.5%	4.1%	1.1%	0.0%	0.0%
Other	0.0%	1.1%	0.7%	0.0%	0.7%	2.2%	1.9%	1.1%	0.4%	0.0%
**Firmicutes**										
Streptococcaceae	18.6%	16.7%	33.1%	26.8%	34.6%	24.9%	8.9%	26.0%	37.5%	82.9%
Veillonellaceae	8.2%	21.6%	8.2%	22.3%	17.5%	7.8%	23.4%	16.4%	15.2%	4.5%
Staphylococcaceae	0.0%	0.0%	0.0%	0.0%	0.0%	0.0%	0.0%	0.0%	0.0%	0.0%
Bacillales Family XI. Incertae Sedis	1.1%	0.0%	1.5%	0.7%	0.0%	3.3%	0.7%	2.6%	0.4%	0.0%
Unclassified Lactobacillales	2.2%	1.5%	4.1%	0.4%	0.4%	5.9%	1.5%	5.9%	7.1%	0.4%
Lachnospiraceae	2.2%	6.7%	0.4%	2.6%	4.8%	2.2%	2.6%	2.2%	1.5%	0.0%
Clostridiales Family XI. Incertae Sedis	0.0%	0.0%	0.0%	0.4%	0.0%	0.4%	0.0%	0.0%	0.0%	0.4%
Peptostreptococcaceae	0.0%	0.7%	0.0%	1.1%	0.0%	0.0%	0.0%	0.0%	0.0%	0.0%
Carnobacteriaceae	0.0%	0.4%	0.0%	0.0%	0.0%	0.0%	0.0%	0.0%	0.4%	0.0%
Other	0.4%	1.5%	0.0%	1.1%	5.2%	0.4%	1.1%	0.7%	0.4%	0.4%
**Fusobacteria**										
Fusobacteriaceae	1.9%	2.6%	3.0%	7.1%	0.0%	12.3%	7.4%	0.7%	1.1%	4.1%
Leptotrichiaceae	1.9%	0.0%	0.0%	1.9%	0.0%	1.9%	3.0%	3.0%	0.4%	0.0%
**Proteobacteria**										
**Betaproteobacteria**										
Neisseriaceae	10.4%	8.2%	17.5%	3.0%	0.0%	21.9%	0.0%	3.3%	1.5%	0.0%
Comamonadaceae	0.0%	0.0%	0.0%	0.0%	0.0%	0.0%	0.0%	0.0%	0.0%	0.0%
Burkholderiales_incertae_sedis	0.4%	0.0%	0.0%	0.0%	0.0%	0.0%	0.0%	0.0%	0.0%	0.0%
Other	0.0%	0.0%	0.0%	0.0%	0.0%	0.0%	0.0%	0.0%	0.0%	0.0%
**Gammaproteobacteria**										
Pasteurellaceae	3.7%	3.7%	5.9%	2.6%	0.0%	5.9%	7.8%	9.3%	23.0%	0.0%
Moraxellaceae	29.0%	0.0%	0.0%	0.0%	0.0%	0.0%	0.0%	0.0%	0.0%	0.0%
Other	0.0%	0.0%	0.0%	0.0%	0.0%	0.0%	0.0%	0.0%	0.0%	0.0%
**Other Proteobacteria**	0.0%	0.4%	0.0%	0.0%	0.0%	0.0%	0.4%	0.0%	0.0%	0.0%
**Other Phyla**	0.4%	0.4%	0.0%	0.0%	0.4%	0.0%	0.4%	0.0%	0.0%	0.0%
